# Targeting the kynurenine pathway: another therapeutic opportunity in the metabolic crosstalk between cancer and immune cells

**DOI:** 10.3389/fonc.2024.1524651

**Published:** 2025-01-22

**Authors:** Irene Kang, George Theodoropoulos, Medhi Wangpaichitr

**Affiliations:** ^1^ Department of Veterans Affairs, Miami VA Healthcare System, Miami, FL, United States; ^2^ South Florida VA Foundation for Research and Education, Miami, FL, United States; ^3^ Department of Surgery, Division of Thoracic Surgery, University of Miami, Miami, FL, United States; ^4^ Sylvester Comprehensive Cancer Center, University of Miami, Miami, FL, United States

**Keywords:** lung cancer, metabolism, immunometabolism, drug resistance, kynurenine, dual inhibitors

## Abstract

The pivotal role of metabolic reprogramming in cancer-related drug resistance, through the tryptophan-catabolized kynurenine pathway (KP), has been particularly underscored in recent research. This pathway, driven by indoleamine 2,3-dioxygenase 1 (IDO1), facilitates immune evasion and promotes tumor progression by fostering an immunosuppressive environment. In Phase III investigation of the combination of IDO1 inhibition with immune checkpoint inhibitors (ICIs), the combination therapy was not efficacious. In this review, we revisit current advances, explore future directions, and emphasize the importance of dual inhibition of the KP rate-limiting enzymes IDO1 and tryptophan 2,3-dioxygenase-2 (TDO2) in appropriate patient populations. We propose that dual inhibition may maximize the therapeutic potential of KP inhibition. Additionally, we delve into the complex cellular interactions in cancer and metabolic dependencies within the tumor microenvironment (TME). Insights from preclinical studies, recent clinical trials, and promising therapeutic combinations will be discussed to elucidate and promote a clear path forward for the direction of KP research into cancer-related outcomes.

## Introduction

Drug resistance in cancer remains a formidable challenge in modern oncology, severely limiting the efficacy of treatments across a wide spectrum of malignancies. Resistance mechanisms are multifaceted, often involving genetic mutations, epigenetic alterations, or modifications in signaling pathways that protect cancer cells from cytotoxic agents. These mechanisms vary from tissue to tissue and within tumor types in the same tissue. Hence, while treatments like chemotherapy, radiation, and immunotherapy initially showed promise, the emergence of resistant tumor cells decreased therapeutic effectiveness.

These developments increase the challenges in the application of therapies to the most appropriate patient cohort, treatment combination, and timing. This challenge is exemplified in cisplatin, one of the most used chemotherapy drugs. Initially effective in treating a variety of solid tumors, including non-small cell lung cancer (NSCLC), ovarian, and bladder cancers, cisplatin’s effectiveness diminishes as tumor cells develop mechanisms to evade its cytotoxic effects. These mechanisms include enhanced DNA repair capabilities, increased efflux of the drug, and profound alterations in cellular metabolism promoting tumor survival (1, 2).

The reprogramming of cellular metabolism was proposed as a common type of tumor resistance decades ago, but the complexities and uniqueness of certain alterations in a tissue specific manner have only been described in the last decade. Altered metabolism has now emerged as a central factor in cancer resistance, particularly as tumors shift metabolic reliance to sustain growth after treatment. Based on these concepts, our lab has extensively investigated and characterized tumor metabolic pathways in hypoxic [hypoxia-inducible factor (HIF1α)] and normoxic (oxidative phosphorylation) conditions (3–5). This work led to the reporting of various metabolic components that tumor cells relied on for survival. One metabolic pathway that gained attention for promoting survival under treatment pressures is the kynurenine pathway (KP) of tryptophan degradation (6, 7). By degrading tryptophan into immunosuppressive metabolites like kynurenine, cancer cells create an environment that suppresses the activity of effector immune cells. This promotes the expansion of regulatory T cells (Tregs) and myeloid-derived suppressor cells (MDSCs) (8–10), enabling cancer cells to escape immune surveillance and contributing to therapy resistance.

This perspective review will explore the latest advances in targeting metabolic crosstalk with a particular focus on inhibiting the tryptophan-catabolized KP. We will discuss why current therapeutic strategies aimed at disrupting this crosstalk often fail and propose ways to enhance anti-tumor immunity and overcome drug resistance, offering a promising pathway for developing new cancer treatments.

## The role of the kynurenine pathway in cancer metabolism

Tryptophan (TRP) is an essential amino acid required for protein synthesis as a precursor to serotonin and melatonin (11, 12). However, the majority of TRP (about 99%) not used for protein synthesis—is broken down via the kynurenine pathway (KP) to generate kynurenine (KYN) (13). The KP pathway involves rate-limiting key enzymes, including indoleamine 2,3-dioxygenase 1 (IDO1), tryptophan 2,3-dioxygenase (TDO), and indoleamine 2,3-dioxygenase 2 (IDO2). The first step in the pathway is the conversion of tryptophan to N-formyl kynurenine by either IDO1, TDO, or IDO2. N-formyl kynurenine is then rapidly converted into kynurenine, which serves as the precursor for several biologically active metabolites, such as kynurenic acid, xanthurenic acid, and anthranilic acid (14).

**Figure 1:**
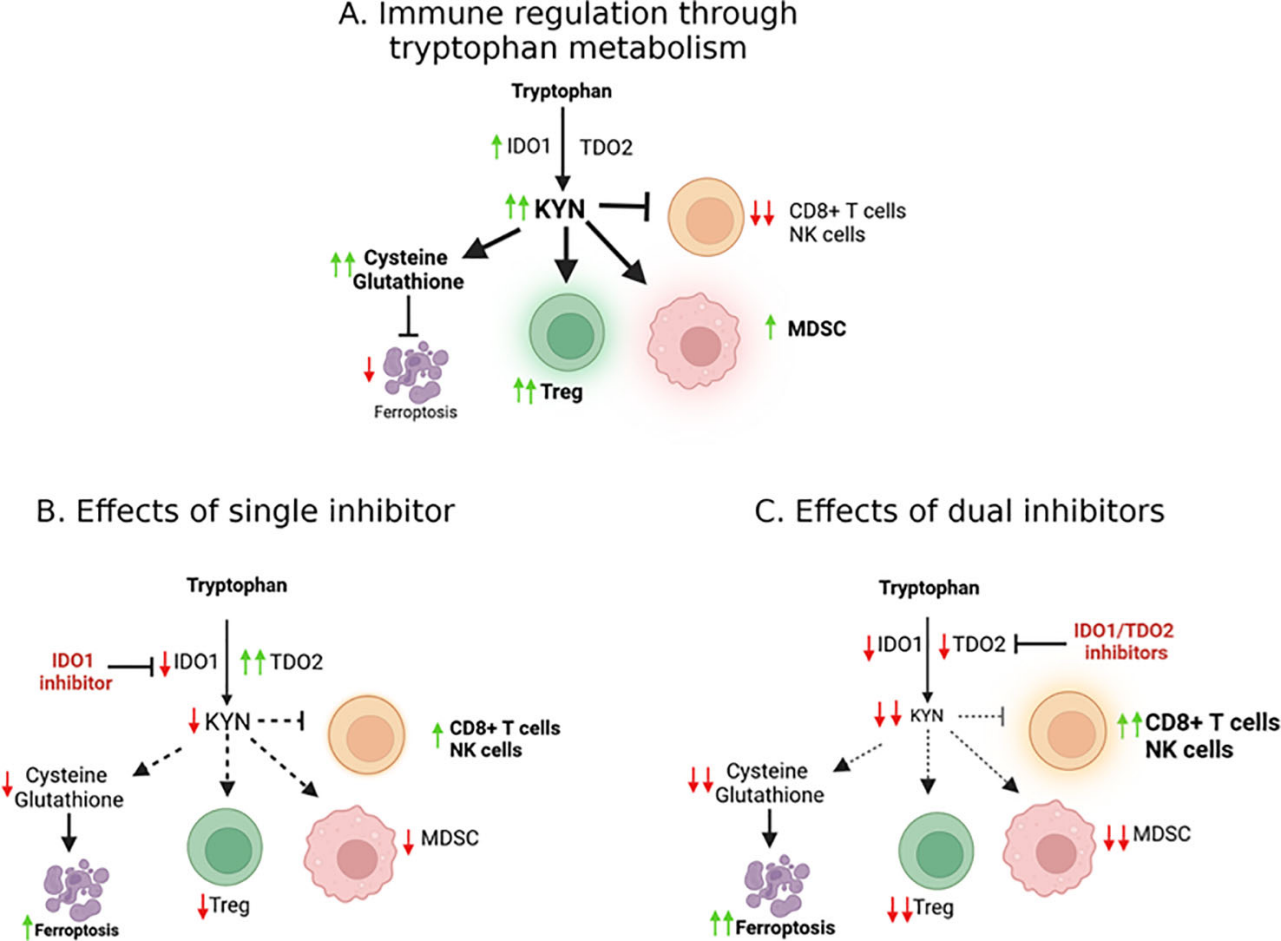
Molecular effects of IDO/TDO signaling. **(A)** The IDO1 enzyme catalyzes the conversion of tryptophan into kynurenine, an oncometabolite. Kynurenine production contributes to an immunosuppressive tumor microenvironment (TME), facilitating cancer progression as well as suppressing ferroptosis. **(B)** TDO2 can potentially compensate when IDO1 is inhibited, suggesting that effective targeting may require simultaneous inhibition of both IDO and TDO to overcome this compensatory mechanism and reduce immunosuppression in the TME **(C)**.

In cancer, IDO1 is overexpressed and has been linked to poor prognosis in several tumor types, including lung, ovarian, and pancreatic cancers (15). Studies also showed significantly shorter survival among patients with high expression of IDO1 or TDO2 (16). The overexpression of IDO1/TDO2 and the consequent accumulation of KYN suppress anti-tumor immunity by promoting the differentiation of Tregs and MDSCs, which in turn, inhibit the activity of effector CD8+T cells and NK cells (**Figure 1A**). We found that the key driver of this inhibition is the level of reactive oxygen species (ROS)-dependent IDO1 activity, rather than IDO1 expression (17). Cisplatin-resistant lung cancer cells possessed higher basal levels of ROS when compared to cisplatin-sensitive cells. Together, this immunosuppressive environment facilitates tumor immune evasion and contributes to the resistance of immunotherapies such as immune checkpoint inhibitors (ICIs).

Beyond its role in immune evasion, the KP also supports cancer cell survival and proliferation by supplying essential metabolic intermediates. KYN can activate signaling pathways that promote cancer cell survival, proliferation, and metastasis. KYN has been shown to activate the aryl hydrocarbon receptor (AHR), a transcription factor involved in cell growth and immune regulation (18). This activation promotes tumor growth, so we investigated inhibition in this context. Indeed, exposure to AHR inhibitors (DMF or CH-223191) resulted in the suppression of IDO1 activities, whereas the addition of KYN increased IDO1 activity in cisplatin-resistant cells (17).

## Kynurenine–hypoxia inducible-1α – aryl hydrocarbon receptor axis

**Figure 2:**
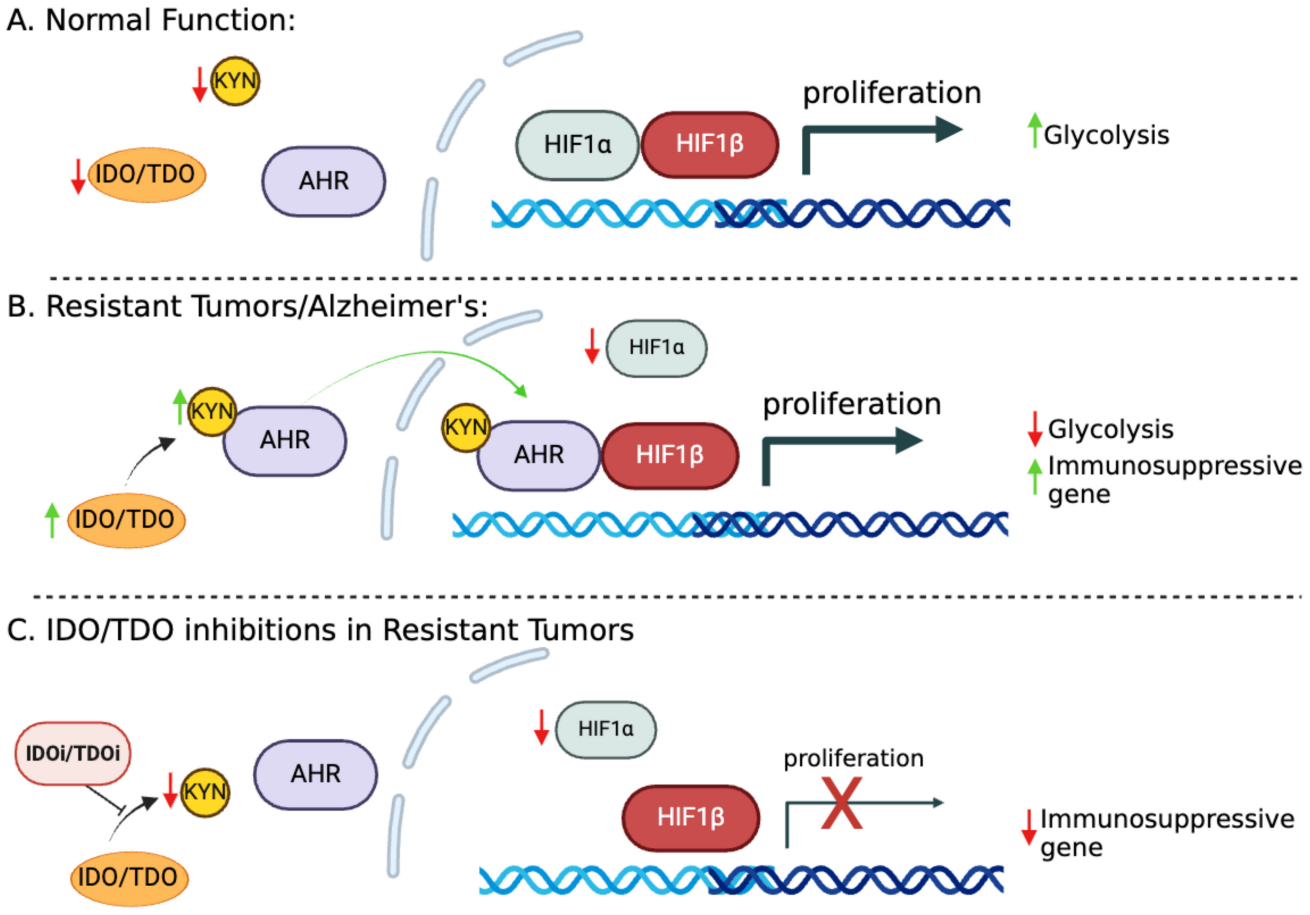
The IDO1-KYN-AHR axis and its role in promoting immunosuppression. **(A)** HIF1α and HIF1β facilitate the transcription of genes essential for glucose metabolism and cell proliferation/survival. **(B)** In cisplatin-resistant cells, increased KYN serves as a ligand for AHR, which becomes activated and translocated to the nucleus. Metabolic reprogramming in resistant cells leads to HIF1α downregulation, allowing ARNT to preferentially bind with activated AHR. This switch to AHR-driven pathways enhances the expression of immunosuppressive genes, fostering tumor immune evasion. **(C)** Targeting KYN could potentially reverse the immunosuppressive tumor microenvironment (TME), offering a therapeutic strategy to enhance antitumor immunity.

ARNT (AHR nuclear translocator) or HIF1β (hypoxia-inducible factor 1β) is a known binding partner of both HIF1α and AHR (**Figure 2**) (19, 20). We reported that HIF1α levels are low in cisplatin-resistant lung cancer cells. Downregulation of HIF1α allows ARNT to preferentially bind with activated AHR rather than HIF1, shifting the metabolic balance towards AHR-driven pathways (17, 21). This pathway enhances immune suppression by increasing FoxP3 (master regulator in the development and function of Tregs) expression and creating an immunosuppressive TME. The extensive characterization of these molecular pathways in lung cancer has led us to promote the concept that the components of these paths could be therapeutic targets helping eliminate resistant cells.

Minhas et al. recently reported that IDO1 is upregulated in response to amyloid and tau pathology in astrocytes, leading to increased production of kynurenine (KYN); these findings are consistent with our model (**Figure 2B**). This upregulation activates AHR and disrupts the balance between AHR and HIF1α signaling in astrocytes, suppressing astrocytic glycolysis and reducing lactate production. Astrocytic glycolysis is essential for neuronal support and disrupting this metabolic pathway contributes to neurodegeneration in Alzheimer’s disease (AD) (22).

In both cancer and neurodegeneration, the IDO1/TDO2-KYN-AHR axis is central to metabolic alterations and immune evasion, highlighting its significance as a therapeutic target. This disruption of normal cellular metabolism and promotion of an immune-suppressive environment makes the KP a possible therapeutic target for both cancerous and neurodegenerative conditions.

## Kynurenine and immune evasion: mechanistic insights

The immune system’s ability to recognize and eliminate tumor cells is a critical component of effective cancer therapy (ICIs and others). In certain patients with tumors that escape immune surveillance, treatment is not effective. One of the central mechanisms by which tumors achieve this immune evasion is through the induction of IDO1 activity and the subsequent depletion of tryptophan and accumulation of kynurenine (**Figure 1A**) (23, 24).

Tryptophan depletion alone has significant effects on immune cell function since T cells, particularly effector T cells, are highly sensitive to tryptophan availability. In low tryptophan conditions, T cell proliferation is inhibited, and cells become functionally anergic. Moreover, kynurenine directly inhibits T cell proliferation and induces the differentiation of naïve T cells into Tregs, further suppressing the immune response (25–27). Tryptophan depletion and the resultant kynurenine accumulation favor immunosuppression over activation in the TME.

IDO1 and kynurenine also promote the expansion of MDSCs, a population of immune cells that suppresses both innate and adaptive immune responses (**Figure 1A**). MDSCs inhibit the activation of effector T cells and NK cells; additionally, they produce high levels of reactive oxygen species (ROS) and nitric oxide, which inhibit T cell receptor signaling and promote tumor progression (28, 29). Higher ROS levels generated by MDSCs may also further enhance IDO1 activity.

## Crosstalk between cancer cells and immune cells in the TME

The metabolic crosstalk between cancer cells and immune cells in the TME is a key determinant of tumor progression and therapy resistance. By reprogramming their metabolism, cancer cells create an environment that is hostile to immune effector cells but supportive of immunosuppressive cells. This crosstalk can be mediated by a variety of metabolic pathways, including glycolysis, glutamine metabolism, and the KP.

In addition to the direct effects of TRP depletion and KYN accumulation on immune cells, cancer cells also engage in metabolic competition with immune cells. Effector T cells, for example, rely on glycolysis to support their rapid proliferation and production of cytokines (30). However, in the nutrient-deprived environment of the TME, cancer cells outcompete T cells for glucose, limiting the availability of this critical nutrient for immune cell function (31–33). Similarly, cancer cells’ reliance on glutamine for the tricarboxylic acid (TCA) cycle also depletes the available glutamine for immune cell activity.

One of the most intriguing aspects of this metabolic crosstalk is the role of kynurenine in the ferroptosis of cancer cells, a form of programmed cell death characterized by the accumulation of lipid peroxides. KYN has an anti-ferroptosis effect that not only supports cancer cell survival but also creates an environment that is resistant to oxidative stress, further promoting immune evasion and drug resistance. Ferroptosis is regulated by the cystine/glutamate antiporter system (xCT), which imports cystine into cells in exchange for glutamate (34). Cystine is then reduced to cysteine, which is required for the synthesis of glutathione, a critical antioxidant that protects cells from ferroptosis. Unlike apoptosis, which is often inhibited in cancer cells, ferroptosis is regulated by the availability of cysteine and the function of the xCT antiporter. By disrupting the cystine/glutamate exchange, cancer cells can be sensitized to ferroptosis, making it an interesting and promising new target for therapy (34, 35).

KYN has been shown to inhibit ferroptosis by upregulating the expression of xCT, thereby enhancing cystine import and glutathione synthesis (34, 36, 37). This protects cancer cells from oxidative stress and ferroptosis (37). By inhibiting the kynurenine pathway, it may be possible to disrupt this protective mechanism and re-sensitize cancer cells to ferroptosis, offering a new avenue for cancer therapy (**Figure 1B**). Preclinical studies have shown that combining ferroptosis inducers with KP inhibitors can enhance the anti-tumor effects of both therapies. In a recent study by Fiore et al., inhibition of IDO1 sensitized cancer cells to ferroptosis, leading to increased cell death *in vitro* and reduced tumor growth *in vivo* (37) by inducing cell death and enhancing the immune response.

## Inhibition of IDO1 and TDO2: a promising therapeutic strategy

The failure of single-agent IDO1 inhibitors, such as epacadostat, in clinical trials highlighted the limitations of targeting a single enzyme in the kynurenine pathway (**Table 1**). One of the key challenges is the compensatory upregulation of other enzymes, such as TDO2, which can maintain kynurenine production in the presence of IDO1 inhibitors (**Figure 1B**) (26, 38). To overcome this challenge, researchers have begun exploring the potential of dual inhibitors targeting both IDO1 and TDO2 (**Figure 1C**), thereby reducing the likelihood of compensatory metabolic pathways sustaining kynurenine production (39, 40).

**Table 1:** IDO1 and TDO therapies in clinical development.

Drug name	Target	Description/Effects
Epacadostat (INCB024360)	IDO1	Studied with ICIs (such as pembrolizumab and nivolumab) for advanced melanoma tumors; failed to meet primary endpoints and trials were suspended
Indoximod (1-methyl-D-tryptophan)	IDO1	Acts as a tryptophan mimetic, evaluated for breast cancer and melanoma (alongside other therapies)
Linrodostat (BMS-986205)	IDO1	Tested in combination with nivolumab for non-small lung, head, and neck cancers; stopped in Phase III trials dur to industry challenges
Navoxiimod (GDC-0919)	IDO1	Assessed in early-phase clinical trials (as a monotherapy and with other agents) for advanced solid tumors
PF-06840003	IDO1	Went through clinical trials for safety and efficacy against advanced malignancies
KHK2455	IDO1	Investigated for potential in treating advanced solid tumors (both along and with other treatments)
M4112	IDO1/TDO2	Demonstrated safety and efficacy as a monotherapy in Phase I trials, though plasma kynurenine levels were not significantly reduced in a steady state
AT-0174	IDO1/TDO2	Showed significant tumor growth suppression in platinum-resistant non-small lung cancer models, especially with anti-PD-1 therapy

Green denotes IDO1 inhibitors. Blue denotes IDO1/TDO2 inhibitors.

In our recent preclinical studies, dual inhibition of IDO1 and TDO2 with the novel agent AT-0174 effectively reduced KYN levels, increased tumor infiltration with natural killer cells, and reduced regulatory T cells in cisplatin-resistant lung cancer models (38). In these studies, dual inhibition of IDO1 and TDO2 as monotherapy was similarly effective on overall survival as anti-PD1 monotherapy. Moreover, AT-0174 was synergistic when combined with anti-PD1 therapy on significant reduction of tumor growth, enhanced infiltration of CD8+ T cells into the TME, and improved survival time in animal models of treatment-resistant NSCLC tumors. This combination therapy not only reduced the immunosuppressive effects of KYN but also promoted immune-mediated tumor clearance.

These mechanisms were further substantiated in an aggressive model of glioblastoma where AT-0174 monotherapy increased natural killer cell infiltration, reduced Treg cells in tumor tissues, and was synergistic with Temozolomide (TMZ), an alkylating agent, in significantly prolonging animal survival (41). Glioblastoma is a highly aggressive brain cancer with limited treatment options and rapid resistance development to TMZ. These results suggest that AT-0174 administered at the time of Temozolomide initiation may, through synergistic mechanisms, aid in immune-mediated elimination of emergent tumor variants with resistance to Temozolomide, thereby improving patient survival.

In another study of high-grade serous carcinoma (HGSC), which is known to exhibit poor outcomes due to therapy resistance and an immunosuppressive TME, Crump et al. reported that HGSC tumors are driven predominantly by TDO2 in promoting tumor progression and immune evasion via kynurenine (KYN) production. High IL6 levels, linked to poor prognosis, correlate with elevated KYN in patient samples. Dual inhibition of IDO1/TDO2 using AT-0174 reduced tumor growth, diminished tumor-associated macrophages (TAMs), and suppressed PD-L1 expression. Cisplatin in combination with AT-0174 extended survival in preclinical models (42).

Together, these studies highlighted the potential of metabolic reprogramming of the TME to overcome immunotherapy resistance and provide a basis for advancing dual IDO1/TDO2 inhibitors in clinical settings.

## Clinical trials

**Figure 3:**
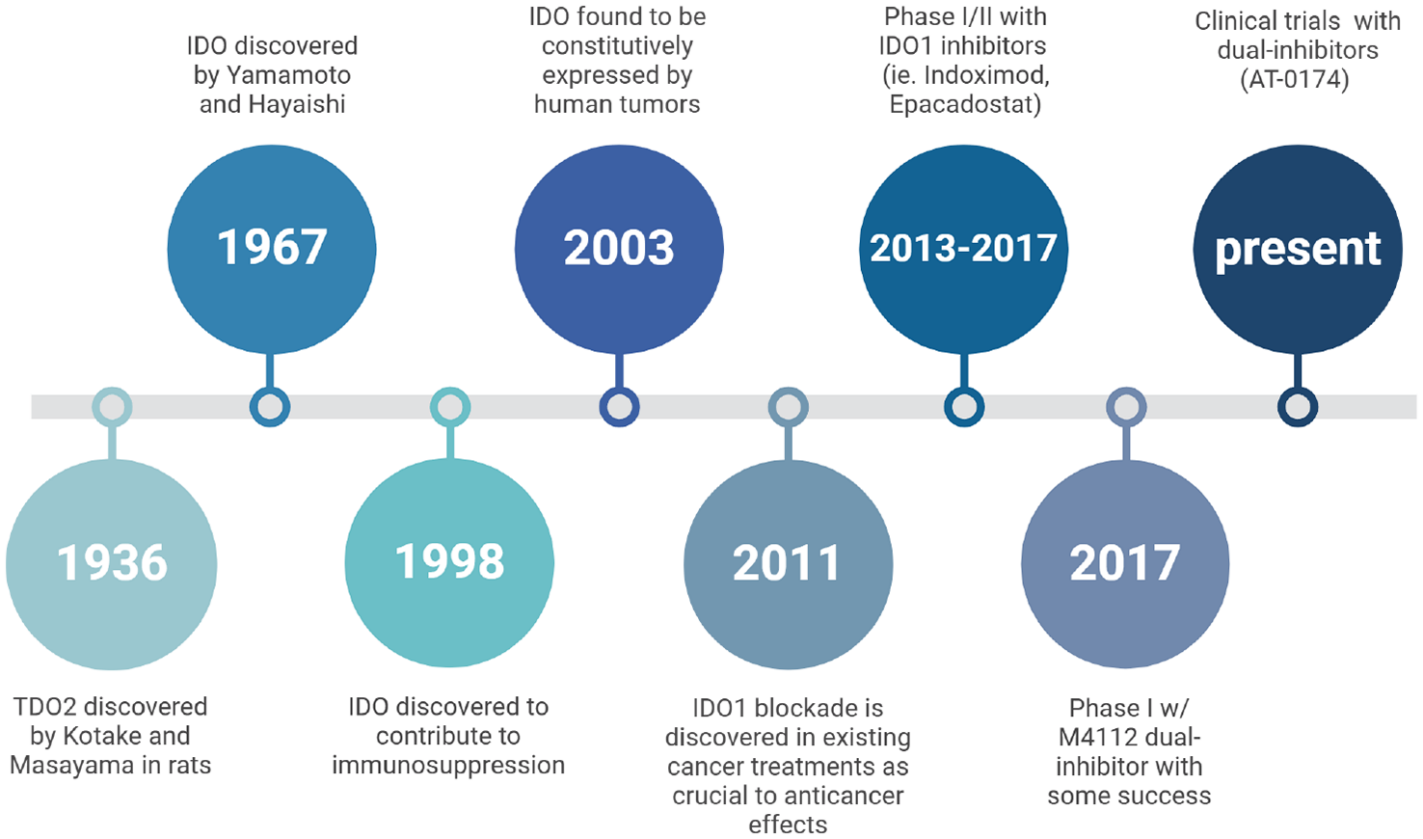
A historical timeline tracing research milestones on the physiology of IDO and TDO. It highlights key discoveries in the understanding of these enzymes’ roles, particularly in immune regulation and cancer, and the development of inhibitors targeting IDO/TDO pathways to explore therapeutic potential.

The first dual IDO1 and TDO2 inhibitor Phase I clinical trial (NCT03306420) was completed with 15 enrolled patients with advanced solid tumors (43). Another potent dual-inhibitor, AT-0174, is currently being tested across multiple sites in an initial clinical trial (ACTRN12623000956606), evaluating the efficacy in patients also with advanced metastatic solid cancers with promising preliminary results (**Figure 3**) (44). M4112, another IDO1/TDO2 inhibitor, did not present any serious safety concerns at doses up to 800 mg twice daily, though the best overall response observed was stable disease in nine patients (60%) with a progression-free survival of 3.7 months; it is noteworthy, however, that most of these patients had tumor types that are typically unresponsive to immunotherapy (43, 45). Unfortunately, due to the early termination of the study, neither the maximum tolerated dose (MTD) nor the recommended phase 2 dose (RP2D) was established. Another limitation of this study was that patient tumor biopsies were not obtained, but now, a KYN antibody is commercially available that could have been used to conduct immunohistochemistry staining. Therefore, neither changes in IDO/TDO expression nor changes in the tumor microenvironment could be evaluated. The termination of this study points to the potential for the appropriate patient cohort to have been investigated, possibly by characterizing patient KYN levels prior to treatment.

While the final results from the *ACTRN* trial are still pending, findings from the *NCT* trial suggest that dual inhibition of IDO1 and TDO2 may enhance the effectiveness of immunotherapies by reversing the immunosuppressive environment created by the KP. Therefore, further research into the pharmacodynamics, safety, and efficacy of dual inhibitors in combination with immune checkpoint inhibitors (ICIs) is of prime importance and warrants further investigation.

## Inhibition of the kynurenine pathway combined with immune checkpoint blockade

Immune checkpoint inhibitors, such as anti-PD1 and anti-CTLA4 antibodies, have revolutionized the treatment of certain cancers by unleashing the immune system to attack tumor cells. However, many patients do not respond to these therapies, particularly those with tumors that have developed mechanisms of immune evasion. One of the primary mechanisms of immune evasion is the upregulation of the kynurenine pathway, which suppresses the activation and proliferation of effector immune cells (10, 46).

By combining KP inhibitors with immune checkpoint blockade, it may be possible to enhance the effectiveness of immunotherapy in these resistant tumors. Inhibiting the KP restores tryptophan levels and reduces the accumulation of immunosuppressive metabolites, thereby allowing effector T cells to proliferate and attack the tumor. Preclinical studies from our group and others have demonstrated that dual inhibition of IDO1 and TDO2, especially when combined with anti-PD1 therapy, can significantly improve survival in mouse models of lung cancer (47, 48). This combination therapy not only reduces tumor growth but also enhances the immune response, leading to durable tumor regression.

## Future directions

While the KP represents a promising therapeutic target, it is unlikely that a one-size-fits-all approach will be effective for all patients. The expression of IDO1, TDO2, and other enzymes in the kynurenine pathway varies widely among different tumor types and even among patients with the same type of cancer (49–52). Testing (e.g. KYN levels) and additional characterization of expression levels in sensitive and resistant tumor types is warranted among all patient populations. Therefore, personalized approaches that tailor therapy based on specific metabolic tumor profiles are likely to be more effective in improving outcomes. Biomarker-driven approaches could help identify patients who are most likely to benefit from KP inhibitors and other combination therapies. Future clinical trials should evaluate the use of new and existing tests to assess biomarkers, such as IDO1/TDO2 expression and kynurenine levels, to guide treatment decisions. Another logical combination treatment could involve the use of AHR inhibitors to suppress IDO1 activities.

While preclinical studies have demonstrated the potential of targeting the KP to overcome drug resistance, translating these findings into clinical practice will require carefully designed trials. One of the key challenges in clinical translation is identifying the optimal combination of therapies that can effectively target the kynurenine pathway while minimizing toxicity. Combining IDO1/TDO2 inhibitors with ICIs has shown promise in preclinical models, but the safety and efficacy of this combination need to be validated in clinical trials.

## Conclusion

Mechanisms of cancer resistance mediated by metabolic alterations point to the kynurenine pathway as a critical metabolic axis in the TME that supports cancer cell survival and immune evasion. By targeting and decreasing the metabolic crosstalk between cancer cells and immune cells, particularly through dual inhibition of IDO1 and TDO2, new therapeutic strategies can be developed to overcome drug resistance and improve patient outcomes. These targets can not only be used to identify tumors that may be sensitive to inhibitors but also offer the potential for combining kynurenine pathway inhibition with immune checkpoint blockade as a promising approach to treating resistant cancers. As research into the metabolic vulnerabilities of cancer cells continues to evolve, the kynurenine pathway will likely remain a key target for future therapeutic interventions.

## Data Availability

The raw data supporting the conclusions of this article will be made available by the authors, without undue reservation.

## References

[1] Galluzzi L Senovilla L Vitale I Michels J Martins I Kepp O. Molecular mechanisms of cisplatin resistance. Oncogene. (2012) 31:1869–83. doi: 10.1038/onc.2011.384 21892204

[2] Muggia FM Bonetti A Hoeschele JD Rozencweig M Howell SB. Platinum antitumor complexes: 50 years since Barnett Rosenberg’s discovery. J Clin Oncol. (2015) 33:4219–26. doi: 10.1200/JCO.2015.60.7481 26503202

[3] Wangpaichitr M Savaraj N Maher J Kurtoglu M Lampidis TJ Intrinsically lower AKT. mammalian target of rapamycin, and hypoxia-inducible factor activity correlates with increased sensitivity to 2-deoxy-D-glucose under hypoxia in lung cancer cell lines. Mol Cancer Ther. (2008) 7:1506–13. doi: 10.1158/1535-7163.MCT-07-2334 PMC2587287 18566221

[4] Maher JC Wangpaichitr M Savaraj N Kurtoglu M Lampidis TJ. Hypoxia-inducible factor-1 confers resistance to the glycolytic inhibitor 2-deoxy-D-glucose. Mol Cancer Ther. (2007) 6:732–41. doi: 10.1158/1535-7163.MCT-06-0407 17308069

[5] Long Y Tsai WB Chang JT Estecio M Wangpaichitr M Savaraj N. Cisplatin-induced synthetic lethality to arginine-starvation therapy by transcriptional suppression of ASS1 is regulated by DEC1, HIF-1alpha, and c-Myc transcription network and is independent of ASS1 promoter DNA methylation. Oncotarget. (2016) 7:82658–70. doi: 10.18632/oncotarget.12308 PMC5347722 27765932

[6] Gouasmi R Ferraro-Peyret C Nancey S Coste I Renno T Chaveroux C. The kynurenine pathway and cancer: why keep it simple when you can make it complicated. Cancers (Basel). (2022) 14:1–15. doi: 10.3390/cancers14112793 PMC9179486 35681770

[7] León-Letelier RA Dou R Vykoukal J Sater AHA Ostrin E Hanash S. The kynurenine pathway presents multi-faceted metabolic vulnerabilities in cancer. Front Oncol. (2023) 13:1256769. doi: 10.3389/fonc.2023.1256769 37876966 PMC10591110

[8] Della Chiesa M Carlomagno S Frumento G Balsamo M Cantoni C Conte R. The tryptophan catabolite L-kynurenine inhibits the surface expression of NKp46- and NKG2D-activating receptors and regulates NK-cell function. Blood. (2006) 108:4118–25. doi: 10.1182/blood-2006-03-006700 16902152

[9] Holmgaard RB Zamarin D Li Y Gasmi B Munn DH Allison JP. Tumor-expressed IDO recruits and activates MDSCs in a treg-dependent manner. Cell Rep. (2015) 13:412–24. doi: 10.1016/j.celrep.2015.08.077 PMC5013825 26411680

[10] Platten M von Knebel Doeberitz N Oezen I Wick W Ochs K. Cancer immunotherapy by targeting IDO1/TDO and their downstream effectors. Front Immunol. (2014) 5:673. doi: 10.3389/fimmu.2014.00673 25628622 PMC4290671

[11] Richard DM Dawes MA Mathias CW Acheson A Hill-Kapturczak N Dougherty DM. L-tryptophan: basic metabolic functions, behavioral research and therapeutic indications. Int J Tryptophan Res. (2009) 2:45–60. doi: 10.4137/IJTR.S2129 20651948 PMC2908021

[12] Szczepanik M. Melatonin and its influence on immune system. J Physiol Pharmacol. (2007) 58:115–24. 18212405

[13] Peters JC. Tryptophan nutrition and metabolism: an overview. Adv Exp Med Biol. (1991) 294:345–58. doi: 10.1007/978-1-4684-5952-4_32 1772073

[14] Badawy AA. Kynurenine pathway of tryptophan metabolism: regulatory and functional aspects. Int J Tryptophan Res. (2017) 10:1178646917691938. doi: 10.1177/1178646917691938 28469468 PMC5398323

[15] Prendergast GC Malachowski WJ Mondal A Scherle P Muller AJ. Indoleamine 2,3-dioxygenase and its therapeutic inhibition in cancer. Int Rev Cell Mol Biol. (2018) 336:175–203. doi: 10.1016/bs.ircmb.2017.07.004 29413890 PMC6054468

[16] Du L Xing Z Tao B Li T Yang D Li W. Both IDO1 and TDO contribute to the Malignancy of gliomas via the Kyn-AhR-AQP4 signaling pathway. Signal Transduct Target Ther. (2020) 5:10. doi: 10.1038/s41392-019-0103-4 32296044 PMC7033114

[17] Nguyen DJM Theodoropoulos G Li YY Wu C Sha W Feun LG. Targeting the kynurenine pathway for the treatment of cisplatin-resistant lung cancer. Mol Cancer Res. (2020) 18:105–17. doi: 10.1158/1541-7786.MCR-19-0239 PMC7262740 31628200

[18] Bessede A Gargaro M Pallotta MT Matino D Servillo G Brunacci C. Aryl hydrocarbon receptor control of a disease tolerance defence pathway. Nature. (2014) 511:184–90. doi: 10.1038/nature13323 PMC4098076 24930766

[19] Denison MS Nagy SR. Activation of the aryl hydrocarbon receptor by structurally diverse exogenous and endogenous chemicals. Annu Rev Pharmacol Toxicol. (2003) 43:309–34. doi: 10.1146/annurev.pharmtox.43.100901.135828 12540743

[20] Semenza GL Wang GL. A nuclear factor induced by hypoxia via *de novo* protein synthesis binds to the human erythropoietin gene enhancer at a site required for transcriptional activation. Mol Cell Biol. (1992) 12:5447–54. doi: 10.1128/mcb.12.12.5447 PMC360482 1448077

[21] Schlichtner S Yasinska IM Klenova E Abooali M Lall GS Berger SM. L-Kynurenine participates in cancer immune evasion by downregulating hypoxic signaling in T lymphocytes. Oncoimmunology. (2023) 12:2244330. doi: 10.1080/2162402X.2023.2244330 37577144 PMC10416736

[22] Minhas PS Jones JR Latif-Hernandez A Sugiura Y Durairaj AS Wang Q. Restoring hippocampal glucose metabolism rescues cognition across Alzheimer’s disease pathologies. Science. (2024) 385:eabm6131. doi: 10.1126/science.abm6131 39172838 PMC12313320

[23] Zhai L Bell A Ladomersky E Lauing KL Bollu L Sosman JA. Immunosuppressive IDO in cancer: mechanisms of action, animal models, and targeting strategies. Front Immunol. (2020) 11:1185. doi: 10.3389/fimmu.2020.01185 32612606 PMC7308527

[24] Passarelli A Pisano C Cecere SC Di Napoli M Rossetti S Tambaro R. Targeting immunometabolism mediated by the IDO1 Pathway: A new mechanism of immune resistance in endometrial cancer. Front Immunol. (2022) 13:953115. doi: 10.3389/fimmu.2022.953115 36119020 PMC9479093

[25] Rad Pour S Morikawa H Kiani NA Yang M Azimi A Shafi G. Exhaustion of CD4+ T-cells mediated by the kynurenine pathway in melanoma. Sci Rep. (2019) 9:12150. doi: 10.1038/s41598-019-48635-x 31434983 PMC6704156

[26] Stone TW Williams RO. Modulation of T cells by tryptophan metabolites in the kynurenine pathway. Trends Pharmacol Sci. (2023) 44:442–56. doi: 10.1016/j.tips.2023.04.006 37248103

[27] Kim M Tomek P. Tryptophan: A rheostat of cancer immune escape mediated by immunosuppressive enzymes IDO1 and TDO. Front Immunol. (2021) 12:636081. doi: 10.3389/fimmu.2021.636081 33708223 PMC7940516

[28] Huang J Zhao Y Zhao K Yin K Wang S. Function of reactive oxygen species in myeloid-derived suppressor cells. Front Immunol. (2023) 14:1226443. doi: 10.3389/fimmu.2023.1226443 37646034 PMC10461062

[29] Ohl K Tenbrock K. Reactive oxygen species as regulators of MDSC-mediated immune suppression. Front Immunol. (2018) 9:2499. doi: 10.3389/fimmu.2018.02499 30425715 PMC6218613

[30] Cao J Liao S Zeng F Liao Q Luo G Zhou Y. Effects of altered glycolysis levels on CD8(+) T cell activation and function. Cell Death Dis. (2023) 14:407. doi: 10.1038/s41419-023-05937-3 37422501 PMC10329707

[31] Chang CH Qiu J O’Sullivan D Buck MD Noguchi T Curtis JD. Metabolic competition in the tumor microenvironment is a driver of cancer progression. Cell. (2015) 162:1229–41. doi: 10.1016/j.cell.2015.08.016 PMC4864363 26321679

[32] Cadenas-De Miguel S Lucianer G Elia I. The metabolic cross-talk between cancer and T cells. Trends Biochem Sci. (2023) 48:597–609. doi: 10.1016/j.tibs.2023.03.004 37080875

[33] Yin Z Bai L Li W Zeng T Tian H Cui J. Targeting T cell metabolism in the tumor microenvironment: an anti-cancer therapeutic strategy. J Exp Clin Cancer Res. (2019) 38:403. doi: 10.1186/s13046-019-1409-3 31519198 PMC6743108

[34] Koppula P Zhuang L Gan B. Cystine transporter SLC7A11/xCT in cancer: ferroptosis, nutrient dependency, and cancer therapy. Protein Cell. (2021) 12:599–620. doi: 10.1007/s13238-020-00789-5 33000412 PMC8310547

[35] Dixon SJ Patel DN Welsch M Skouta R Lee ED Hayano M. Pharmacological inhibition of cystine-glutamate exchange induces endoplasmic reticulum stress and ferroptosis. Elife. (2014) 3:e02523. doi: 10.7554/eLife.02523 24844246 PMC4054777

[36] Lin W Wang C Liu G Bi C Wang X Zhou Q. SLC7A11/xCT in cancer: biological functions and therapeutic implications. Am J Cancer Res. (2020) 10:3106–26. PMC7642655 33163260

[37] Fiore A Zeitler L Russier M Groß A Hiller MK Parker JL. Kynurenine importation by SLC7A11 propagates anti-ferroptotic signaling. Mol Cell. (2022) 82:920–932.e7. doi: 10.1016/j.molcel.2022.02.007 35245456 PMC7617904

[38] Wu C Spector SA Theodoropoulos G Nguyen DJM Kim EY Garcia A. Dual inhibition of IDO1/TDO2 enhances anti-tumor immunity in platinum-resistant non-small cell lung cancer. Cancer Metab. (2023) 11:7. doi: 10.1186/s40170-023-00307-1 37226257 PMC10207715

[39] Peng X Zhao Z Liu L Bai L Tong R Yang H. Targeting indoleamine dioxygenase and tryptophan dioxygenase in cancer immunotherapy: clinical progress and challenges. Drug Des Devel Ther. (2022) 16:2639–57. doi: 10.2147/DDDT.S373780 PMC9374094 35965963

[40] Yoshioka S Ikeda T Fukuchi S Kawai Y Ohta K Murakami H. Identification and characterization of a novel dual inhibitor of indoleamine 2,3-dioxygenase 1 and tryptophan 2,3-dioxygenase. Int J Tryptophan Res. (2022) 15:11786469221138456. doi: 10.1177/11786469221138456 36467776 PMC9716449

[41] Bickerdike MJ Nafia I Bessede A Chen CB Wangpaichitr M. AT-0174, a novel dual IDO1/TDO2 enzyme inhibitor, synergises with temozolomide to improve survival in an orthotopic mouse model of glioblastoma. BMC Cancer. (2024) 24:889. doi: 10.1186/s12885-024-12631-w 39048947 PMC11267968

[42] Crump LS Floyd JL Kuo LW Post MD Bickerdike M O’Neill K. Targeting tryptophan catabolism in ovarian cancer to attenuate macrophage infiltration and PD-L1 expression. Cancer Res Commun. (2024) 4:822–33. doi: 10.1158/2767-9764.CRC-23-0513 PMC10946310 38451784

[43] Naing A Eder JP Piha-Paul SA Gimmi C Hussey E Zhang S. Preclinical investigations and a first-in-human phase I trial of M4112, the first dual inhibitor of indoleamine 2,3-dioxygenase 1 and tryptophan 2,3-dioxygenase 2, in patients with advanced solid tumors. J Immunother Cancer. (2020) 8:1–10. doi: 10.1136/jitc-2020-000870 PMC7449315 32843490

[44] AntidoTherapeutics, A phase I study to evaluate the safety, tolerability, pharmacology, and preliminary efficacy of AT-0174 in subjects with advanced solid Malignancies(2024). Available online at: https://trials.cancervic.org.au/details/vctl_actrn12623000956606 (Accessed February 20, 2024).

[45] Ventola CL. Cancer immunotherapy, part 3: challenges and future trends. P T. (2017) 42:514–21. PMC5521300 28781505

[46] Jennings MR Munn D Blazeck J. Immunosuppressive metabolites in tumoral immune evasion: redundancies, clinical efforts, and pathways forward. J Immunother Cancer. (2021) 9:1–19. doi: 10.1136/jitc-2021-003013 PMC8527165 34667078

[47] Opitz CA Somarribas Patterson LF Mohapatra SR Dewi DL Sadik A Platten M. The therapeutic potential of targeting tryptophan catabolism in cancer. Br J Cancer. (2020) 122:30–44. doi: 10.1038/s41416-019-0664-6 31819194 PMC6964670

[48] Fujiwara Y Kato S Nesline MK Conroy JM DePietro P Pabla S. Indoleamine 2,3-dioxygenase (IDO) inhibitors and cancer immunotherapy. Cancer Treat Rev. (2022) 110:102461. doi: 10.1016/j.ctrv.2022.102461 36058143 PMC12187009

[49] Hoffmann I Dragomir MP Monjé N Keunecke C Kunze CA Schallenberg S. Increased expression of IDO1 is associated with improved survival and increased number of TILs in patients with high-grade serous ovarian cancer. Neoplasia. (2023) 44:100934. doi: 10.1016/j.neo.2023.100934 37703626 PMC10502412

[50] Meireson A Devos M Brochez L. IDO expression in cancer: different compartment, different functionality? Front Immunol. (2020) 11:531491. doi: 10.3389/fimmu.2020.531491 33072086 PMC7541907

[51] Bessede A Peyraud F Le Moulec S Cousin S Cabart M Chomy F. Upregulation of indoleamine 2,3-dioxygenase 1 in tumor cells and tertiary lymphoid structures is a hallmark of inflamed non-small cell lung cancer. Clin Cancer Res. (2023) 29:4883–93. doi: 10.1158/1078-0432.CCR-23-1928 PMC10690088 37756581

[52] Fujiwara Y Kato S Nishizaki D Miyashita H Lee S Nesline MK. High indoleamine 2,3-dioxygenase transcript levels predict better outcome after front-line cancer immunotherapy. iScience. (2024) 27:109632. doi: 10.1016/j.isci.2024.109632 38632994 PMC11022045

